# Functionality of lyophilized osteoinductive EVs: a mechanistic study

**DOI:** 10.3389/fbioe.2024.1452428

**Published:** 2024-10-22

**Authors:** Chun-Chieh Huang, Miya Kang, Koushik Debnath, Kasey Leung, Vidhath Raghavan, Yu Lu, Lyndon F. Cooper, Praveen Gajendrareddy, Sriram Ravindran

**Affiliations:** ^1^ Department of Oral Biology, University of Illinois Chicago, Chicago, IL, United States; ^2^ Department of Periodontics, University of Illinois Chicago, Chicago, IL, United States; ^3^ School of Dentistry, Virginia Commonwealth University, Richmond, VA, United States

**Keywords:** lyophilization of EVs, engineered extracellular vesicles, mesenchymal stem cells, bone regeneration, BMP2 signaling

## Abstract

**Introduction:**

Mesenchymal stem cell-derived extracellular vesicles (MSC EVs) hold significant promise for regenerative medicine. Lyophilization of EVs significantly enhances their translational potential. While, lyophilized EVs have been studied from a morphological perspective, the functional stability of these EVs and their cargo following lyophilization need to be mechanistically investigated.

**Methods:**

In this study, we investigated the functional and mechanistic bioactivity of fresh versus lyophilized MSC EVs, specifically focusing on functionally engineered osteoinductive EVs developed in our laboratory. We utilized dimethyl sulfoxide (DMSO) as a cryoprotectant and conducted pathway-specific *in vitro* and *in vivo* experiments to assess the stability and functionality of the EVs.

**Results:**

Our findings show that using DMSO as a cryoprotectant before lyophilization preserves the functional stability of engineered MSC EVs. *In vitro* experiments demonstrated that the endocytosis, cargo integrity, and pathway-specific activity of lyophilized EVs were maintained when DMSO was used as the cryoprotectant. Additionally, *in vivo* bone regeneration studies revealed that the functionality of cryoprotected lyophilized EVs was comparable to that of freshly isolated EVs.

**Discussion:**

These results provide a foundation for evaluating the functionality of lyophilized EVs and exploring the use of DMSO and other cryoprotectants in EV-based therapies. Understanding the functionality of lyophilized naïve and engineered EVs from a mechanistic perspective may enhance validation approaches for tissue regeneration strategies.

## Introduction

Extracellular vesicles (EVs) are nanoscale vesicles secreted by both prokaryotic and eukaryotic cells (Mobarak et al., 2024). The role of EVs in regenerative medicine has gained prominence recently owing to their unique properties that represent the characteristics of their parental cells. They are mediators of cellular function and influence the activity of target cells in a paracrine manner (Lai et al., 2010; Reis et al., 2012; Mokarizadeh et al., 2012). In particular, mesenchymal stem cell derived extracellular vesicles (MSC EVs) possess immense therapeutic potential (Cooper et al., 2019; Kou et al., 2022). This paracrine activity is attributed to the many of the immunomodulatory and regenerative properties of MSCs (Yao et al., 2015; Dai et al., 2007; Gnecchi et al., 2008). The role of MSC EVs in regenerative medicine and disease therapy has been elucidated by various groups in multiple organ systems as well as in several disease models (Lim et al., 2023; Ma et al., 2022; Weng et al., 2021).

Our laboratory has studied various mechanistic aspects of MSC EVs and identified key properties that govern their function. We showed that MSC EVs are endocytosed by their recipient cells via the heparan sulfate proteoglycan (HSPG) receptors on the cell surface mediated by the caveolar endocytic pathway (Huang et al., 2020a; Huang et al., 2016). When MSCs were differentiated or treated with immunomodulatory cytokines, their respective EVs gained lineage-specific properties (Huang et al., 2020a; Kang et al., 2022). Using Dicer and Argonaute knockdown in MSCs, we showed that the miRNA composition of MSC EVs was important for its functionality (Shirazi et al., 2021). Based on these foundational studies, we have devised mechanisms to engineer MSC EVs for enhanced immunomodulatory and regenerative functions (Huang et al., 2023; Huang et al., 2020b; Mathew et al., 2023). These studies underline the potential of both naïve and engineered MSC EVs in medicine.

While the potential is immense, clinical translation of MSC EVs faces several challenges such as isolation procedures, purity, batch variation and long term stability (Gowen et al., 2020). Although isolation can be overcome by precipitation-based techniques and purity and batch variation by the use of cell lines like we showed in our publications with engineered EVs (Huang et al., 2023; Huang et al., 2020b; Mathew et al., 2023; Huang et al., 2021), one major concern about storage and stability remains understudied. Lyophilization/freeze drying is one way to ensure long-term stability. This method has been successfully used for proteins with FDA approved rhBMP2. However, EVs contain a phospholipid bilayer that is similar to the plasma membrane of the parental cells that encapsulates various proteins, miRNA, mRNA cargo, all essential for EV functionality. Freezing of EVs without a cryoprotectant may result in damage to the fragile EV membrane resulting in loss of functionality of the EVs either by preventing endocytosis of the damaged EV or by loss of cargo from the compromised EV membrane. Damage to the EV membrane can also occur during the dehydration phase.

Published studies on EV lyophilization have evaluated various cryoprotectants such as sucrose (Trenkenschuh et al., 2022), trehalose, dimethyl sulfoxide and mannitol (Kang et al., 2024). These studies focused on comparing the EV particle size, electron microscopy-based characterizations, zeta potential and miRNA composition in some cases between fresh and lyophilized EVs. However, these characteristics are also shared by EVs lyophilized without a cryoprotectant. Conversely, there has been minimal characterization of EV functionality mechanistically *in vitro* and more importantly *in vivo*. Further, there is presently a significant knowledge gap as to the functionality of lyophilized engineered EVs designed to target specific molecular pathways. The intended effects of these EVs can be realized only if their lyophilized forms are just as effective as freshly isolated EVs. In the present study, based on our foundational studies on EV functionality and using MSC EVs specifically engineered for their ability to induce bone regeneration via augmenting the BMP2 signaling cascade, we evaluated the functionality of lyophilized EVs mechanistically. We have focused on the osteoinductive function of these EVs on human bone marrow derived mesenchymal stem cells (HMSCs) for *in vitro* characterization and their ability to regenerate bone *in vivo*.

## Materials and methods

### Cell culture

Primary human bone marrow derived MSCs (HMSCs) were purchased from Lonza and cultured in αMEM basal media (Gibco) supplemented with 20% fetal bovine serum (FBS, Gibco), 1% (v/v) L-Glutamine (Gibco) and 1% (v/v) antibiotic-antimycotic solution (Gibco). For osteogenic differentiation, HMSCs were cultured in αMEM growth medium containing 100 μg/mL ascorbic acid (Sigma), 10 mM β-glycerophosphate (Sigma), and 10 mM dexamethasone (Sigma).

### BMP2 overexpressing HMSCs

HMSCs constitutively expressing BMP2 used in this study have been previously characterized (Huang et al., 2020b). Briefly, HMSCs were transduced with lentiviral particles containing a BMP2 expression plasmid under the control of EF1α promoter and BMP2 expressing cells were selected for stable expression using puromycin.

### Isolation of EVs

Functionally engineered EVs (FEEs) were isolated from the BMP2 expressing HMSCs as per standardized protocols using a PEG-based isolation reagent (Huang et al., 2020a; Huang et al., 2016; Umar et al., 2024). Briefly, HMSCs were washed with phosphate-buffered saline (PBS) and cultured for 24 h in serum free basal medium. The serum free basal medium was harvested and centrifuged at 3,000 g to remove cellular debris. The medium was then concentrated five-fold (20% of original volume) using a 100 KDa cutoff spin filter (Millipore) and EVs were harvested from this concentrated medium using the isolation reagent at 1x concentration followed by overnight incubation at 4^o^C and subsequent centrifugation at 1,500 g for 30 min.

### Preparation and characterization of lyophilized EVs

The EV lyophilization was performed by adding 0–10% (v/v) DMSO cytoprotectant into the EV suspension with PBS. The EV solutions were frozen at −80°C overnight and dried at −55°C at a pressure of 40Pa for 2 days on a countertop lyophilizer (Heto). The lyophilized EVs were stored at room temperature in a sterile vial until used. The reconstitution of the EV solutions was conducted by adding sterile deionized water (Gibco) equal to the original PBS volume before lyophilization.

The lyophilized EVs were characterized for the presence of EV tetraspanin markers CD63 (1/250, Abcam), CD81 (1/250, Abcam) and intra EV marker TSG101 (1/250, Abcam) by immunoblotting. Cell lysates were used as comparative controls. Nano particle tracking analysis (NTA) was performed to obtain the size distribution of the EVs and transmission electron microscopy was performed to evaluate them morphologically.

The effects of lyophilization on EV functionality was further characterized by comparing the presence of osteoinductive miRNAs in fresh and lyophilized BMP2 EVs. The miRNA was isolated from EVs using Direct-zol RNA MicroPrep kit (Zymo Research) and miRNA concentration was measured using Qubit microRNA assay kit (Thermo Fisher Scientific) and Qubit 4 Fluorometers (Thermo Fisher Scientific). cDNA synthesis was performed using miRNA 1st-Strand cDNA Synthesis kit (Agilent Technologies) with equal amount of miRNA from fresh BMP2 EVs and lyophilized BMP2 EVs. qRT-PCR was performed using miRNA QPCR Master Mix (Agilent Technologies) and custom primers for the selected miRNA (Table 1).

**TABLE 1:** List of primers used.

Genes	Primer sequence	
	Forward (5'- 3′)	Reverse (5'- 3′)
Human GAPDH	CAG​GGC​TGC​TTT​TAA​CTC​TGG	TGG​GTG​GAA​TCA​TAT​TGG​AAC​A
Human RUNX2	TGG​TTA​CTG​TCA​TGG​CGG​GTA	TCT​CAG​ATC​GTT​GAA​CCT​TGC​TA
Human BMP2	ACT​ACC​AGA​AAC​GAG​TGG​GAA	GCA​TCT​GTT​CTC​GGA​AAA​CCT
Human OSX	CCT​CTG​CGG​GAC​TCA​ACA​AC	AGC​CCA​TTA​GTG​CTT​GTA​AAG​G
Human OCN	CAC​TCC​TCG​CCC​TAT​TGG​C	CCC​TCC​TGC​TTG​GAC​ACA​AAG

miRNA	Primer Sequence	
hsa-miR-15a-5p	GGG​TAG​CAG​CAC​ATA​GTT​TGT​G	
hsa-miR-15b-5p	GGG​TAG​CAG​CAC​ATC​ATG​GTT​TAC​A	
hsa-miR-16–5p	GGT​AGC​AGC​ACG​TAA​ATA​TTG​GCG	
has-miR-497–5p	CAG​CAG​CAC​ACT​GTG​GTT​TGT	

### Endocytosis experiments

For endocytosis experiments, EVs were fluorescently labeled using the ExoGlow green labeling kit (System Biosciences) as per manufacture’s protocol. Briefly, the EVs were resuspended in PBS and incubated with the recommended concentration of the labeling reagent at 37°C for 20 min. Following incubation, EVs were isolated using our standardized protocols (Huang et al., 2020a; Huang et al., 2016; Umar et al., 2024). For quantitative experiments, HMSCs were plated in 96 well cell culture plates at a concentration of 10,000 cells per well and incubated for 18 h to facilitate cell attachment. The cells were then incubated with increasing amounts of fluorescently labeled EVs for 2 h at 37°C and subsequently washed with PBS and fixed in 10% neutral buffered formalin. The fluorescence from the endocytosed EVs was measured using a BioTek Cytation 96 well plate reader equipped with the appropriate filter sets to measure green fluorescence. The results were plotted as mean ± SD normalized fluorescence intensities (normalized to background and no EV fluorescence). For qualitative experiments, HMSCs (50,000 cells/well) were seeded onto glass coverslips in 12 well cell culture plates and incubated with fluorescently labeled EVs (1 × 10^5^ EV particles per cell) for 2 h at 37°C. The cells were then fixed, counter stained for actin using phalloidin TRITC (Sigma) and imaged using a Zeiss LSM710 confocal microscope.

For endocytosis blocking experiments, the cells were plated in 96 well cell culture plates as described above. Prior to EV treatment, the EVs were pre-treated with the blocking agent heparin (10 μg/mL, Sigma) for 1 h to block the heparin sulfate proteoglycan binding sites on the EV membrane. Alternatively, cells were incubated at 4°C for 30 min before EV treatments. Fluorescence measurement and quantitation were performed as described in the endocytosis experiment (n = 6 per group).

### Functional studies of lyophilized EVs

For *in vitro* osteogenesis differentiation experiments, 50,000 HMSCs were seeded in 12 well cell culture plates in quadruplicates and 5 × 10^9^ fresh or lyophilized BMP2 EV particles was added to each well. RNA was isolated from the cells and qRT-PCR for osteoinductive genes (Table 1) was performed at 3 and 6 days post EV treatment. Fold change in gene expression was calculated using the ddCt method with respect to cells similarly treated but without EVs.

For the reporter assay, HMSCs were seeded in 24 well cell culture plates in quadruplicates (25,000 cells per well) and transfected with BMP2 pathway specific luciferase reporter plasmid (SBE12 ^18^) using lipofectamine transfection reagent as per previously published protocols (Huang et al., 2020b). Forty-8 hours post transfection, the cells were treated with 250 ng/mL recombinant human BMP2 (rhBMP2), 2.5 × 10^9^ fresh or lyophilized BMP2 EVs for 48 h. Total protein was extracted from the cells and the concentration was determined by BCA assay kit (Thermo Fisher Scientific). Luciferase activity from equal amount of protein from each group was measured using a reporter kit (Promega) and normalized to control. The data is represented as mean % increase ± SD in luciferase activity with respect to control cells. Similarly for the alkaline phosphatase (ALP) assay, HMSCs were seeded in 24 well cell culture plates and cells were cultured in osteogenic differentiation medium along with EVs. Samples were collected at 3 and 7 days and ALP activity was measured using ALP assay kit (Abcam) as per the manufacturer’s protocol (n = 4 per group).

For quantitative analysis of SMAD phosphorylation, an in-cell western was performed. For this experiment, HMSCs were seeded in 96 well cell culture plates (10,000 cells per well) and 1 × 10^9^ EV were added to each well (n = 6). Four hours post treatment, the cells were fixed, permeabilized and immunostained for phospho-SMAD 1/5/8 (1/250, Abcam) and tubulin (1/2000, Thermo Fisher Scientific). The wells were imaged using a Licor Odyssey CLX imager and the fluorescence intensity within each well was quantitated using the image analysis software (Image Studio) provided with the instrument. The results were normalized to tubulin expression.

### EV Binding studies

Quantitative binding experiments were performed in 96 well assay plates coated with type I collagen (5 μg/well, BD Biosciences), or fibronectin (5 μg/well, Sigma) as per published and standardized protocols (Huang et al., 2021). Increasing dose of fluorescently labeled EVs were added (n = 6 per group) and incubated for 2 h at room temperature. The wells were washed 3 times with PBS, fixed in 10% neutral buffered formalin and the fluorescence from the bound EVs was measured using a fluorescent plate reader (BioTek). For qualitative experiments, HMSCs were seeded on to glass coverslips and grown to confluence. They were then subjected to decellularization procedure as per previously published protocols (Huang et al., 2021; Huang et al., 2018; Ravindran et al., 2012; Ravindran et al., 2014) and incubated fluorescently labeled EVs for 2 h, fixed and immunostained with type I collagen (1/100 Sigma) or fibronectin (1/100, Abcam) antibodies and imaged using a Zeiss LSM710 confocal microscope.

### *In vivo* rat calvarial bone defect model

All animal experiments were performed in accordance with protocols approved by the UIC animal care committee (ACC, Assurance no: A3460.01). Briefly, Sprague Dawley rats (220g–270 g) were anesthetized intraperitoneally using Ketamine (80–100 mg/kg)/Xylazine (10 mg/kg). Using aseptic technique, a vertical incision was made in the head at the midline to expose the calvarial bone. The connective tissue was removed and two 5 mm calvarial defects were created bilaterally in the calvarium without dura perforation using a trephine burr. Each defect received either PBS, rhBMP2 or EV suspension containing 1.25 × 10^9^ fresh or lyophilized EVs in a type I collagen sponge (Zimmer). All groups contained 4 animals per group. Eight weeks post-surgery, the rats were euthanized by carbon dioxide asphyxiation and cervical dislocation. The calvaria were dissected, fixed in 10% neutral buffered formalin and the microcomputed tomography (μCT) was performed using a Scanco μCT 50 scanner. The data from the scan was quantitatively analyzed using a custom MatLab software to obtain %BV/TV. The samples were then decalcified in 10% (w/v) EDTA solution, embedded in paraffin, and 10 μm sections were subjected to H&E staining and imaged using a Zeiss Axiovert inverted microscope.

### Statistical analysis

The normal distribution of the data obtained from the experiments was evaluated using the Shapiro-Wilk test. For experiments involving two groups, Student’s t-test with a confidence interval of 95% was utilized. For the experiments involving comparison of more than two groups, One-way ANOVA was performed with a confidence interval of 95%. Pairwise comparisons were performed using Tukey’s *ad hoc* test with a confidence interval of 95%.

## Results

### Endocytosis of lyophilized EVs

**FIGURE 1:**
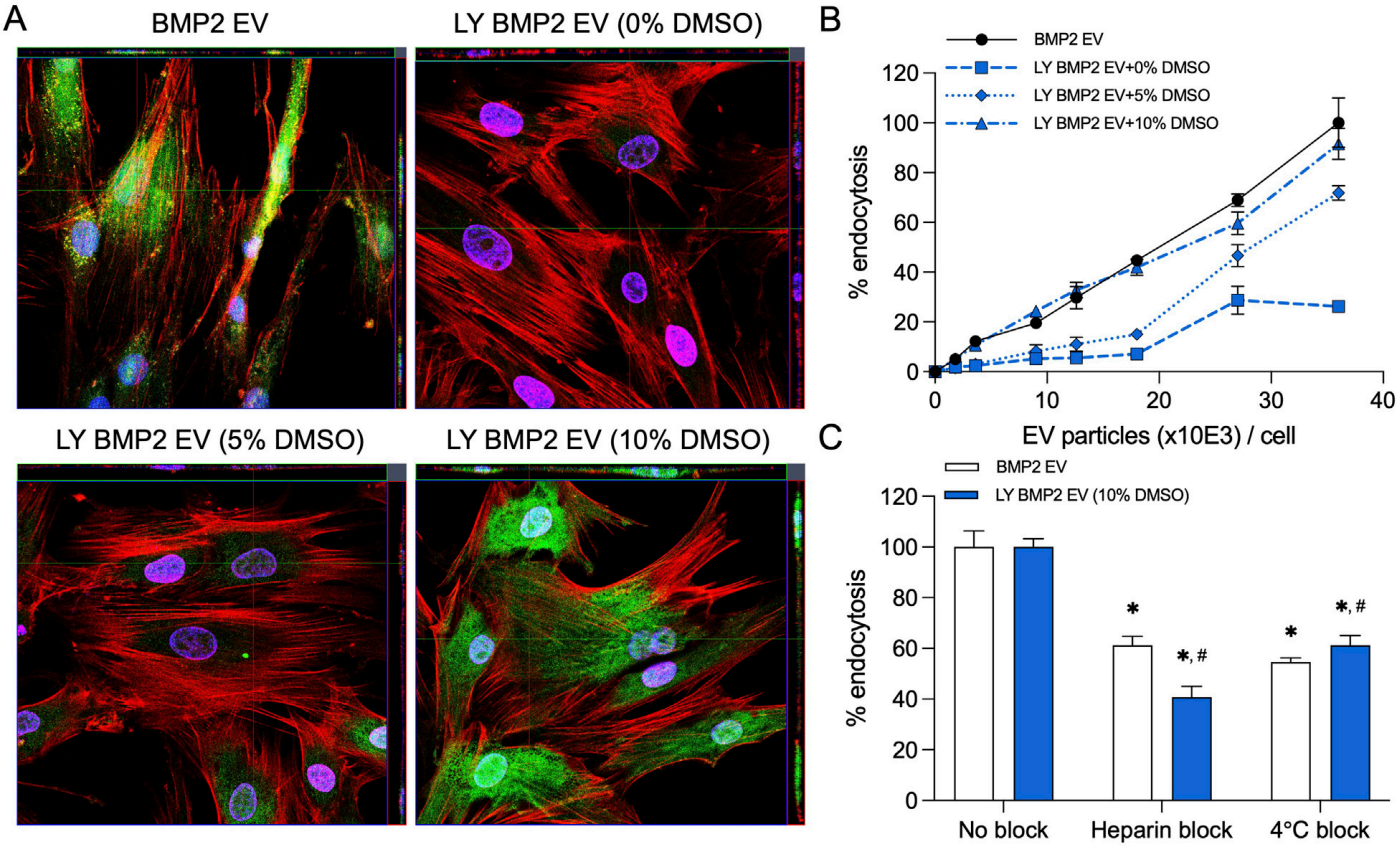
Endocytosis of EVs by HMSCs **(A)** Representative confocal micrograph of fluorescently labeled fresh and lyophilized BMP2 EVs (green) endocytosed by naïve HMSCs at 37°C. Scale bar = 20 μm. **(B)** Dose-dependent endocytosis of fresh and lyophilized BMP2 EVs by HMSCs. Data points represent mean percentage fluorescence ± SD (n = 6). **(C)** Graph showing the inhibition of EV endocytosis after pre-treatment of the EVs with heparin to block interaction with the cell surface HSPGs. The reduction of EV endocytosis at 4°C was also measured compared to 37°C. Data represent mean percentage fluorescence with respect to control ± SD (n = 6). *: statistical significance with respect to No blocking control, #: statistical significance with respect to BMP2 EV group as measured by Student’s t-test (P< 0.05).

MSC EVs are endocytosed by various cells in a dose dependent manner. In prior work, we have shown that endocytosis of the BMP2 EVs by HMSCs is similar to that of naïve MSC EVs. We compared the endocytosis of fresh (BMP2 EVs) and lyophilized EVs (LY BMP2 EVs) qualitatively and quantitatively. Results presented in Figure 1A are representative confocal images of fluorescently labeled BMP2 EVs (green). Fresh BMP2 EVs were readily endocytosed by HMSCs. In lyophilized form, endocytosis was abrogated in EVs that were lyophilized without DMSO. Addition of 5% DMSO resulted in recovery, but not to the level of fresh EVs. With 10% DMSO, the results resembled the endocytosis of fresh EVs qualitatively. Quantitative endocytosis measurements were performed in a dose-dependence assay using fluorescently labeled fresh and lyophilized EVs. Results presented in Figure 1B show that fresh EVs and EVs lyophilized with 10% DMSO showed similar endocytic ability. A significantly reduced endocytic ability was observed in the 5% and no DMSO groups indicating both the dose dependent effect of DMSO as well as the inhibitory effect on the EVs at all doses (n = 6 for all groups and time points). Following these results, we chose to perform functional and morphological characterization of the LY BMP2 EVs that were lyophilized with 10% DMSO and compare them to freshly isolated EV properties.

BMP2 EVs, like MSC EVs, are endocytosed via cell surface HSPGs and require ATP activity for the endocytic process. The presence of heparin and reduction in temperature reduce the efficiency of EV endocytosis (Huang et al., 2020a; Huang et al., 2016). We compared fresh and lyophilized BMP2 EVs for their endocytic pathway specificity. Results presented in Figure 1C show that heparin and reduction in temperature (4^o^C) both significantly reduce the endocytic efficacy of fresh and lyophilized BMP2 EVs. No statistical significance was observed between the BMP2 EV and LY BMP2 EV groups (n = 6 for all groups).

### Electron microscopy of lyophilized EVs

**FIGURE 2:**
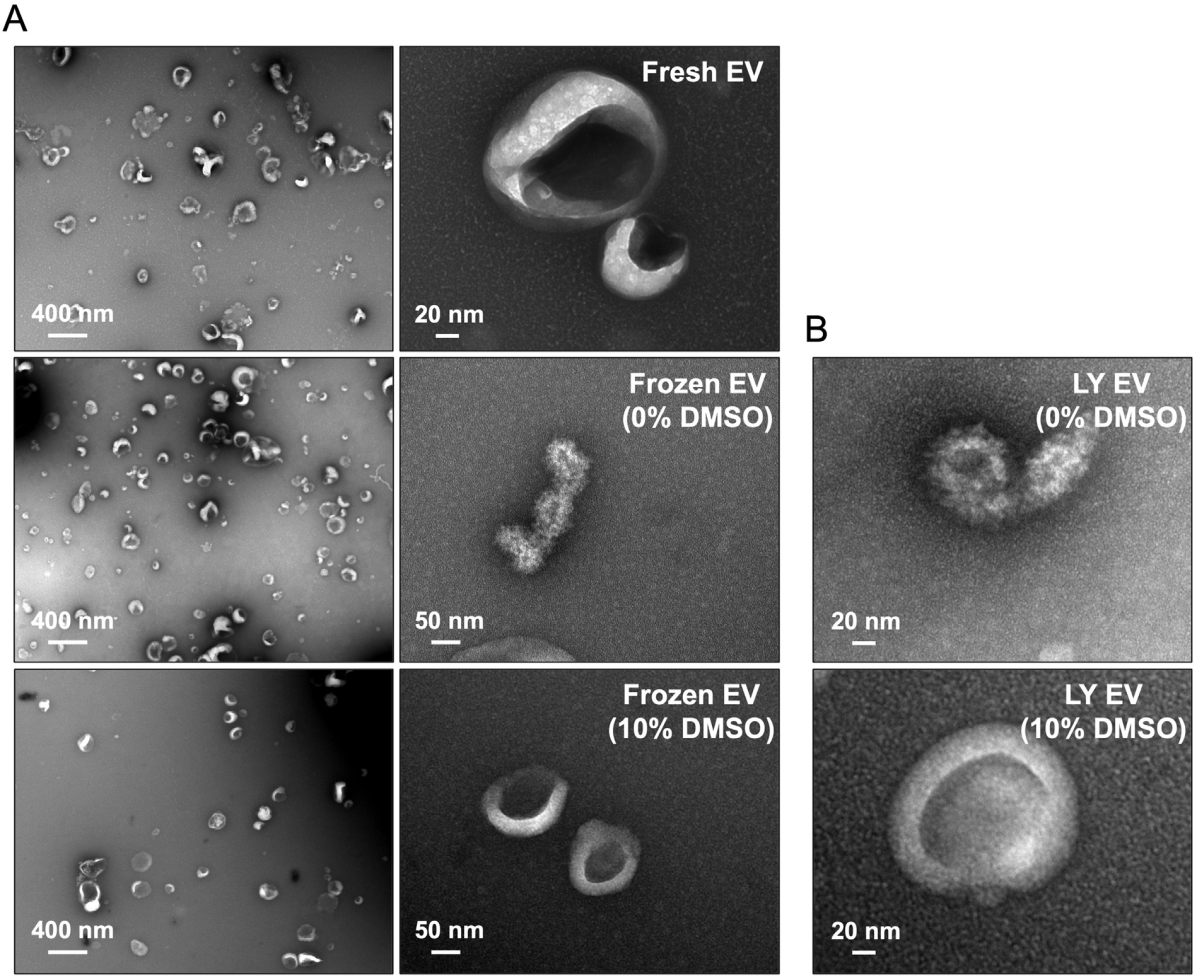
Electron microscopy of lyophilized EVs **(A)** Representative transmission electron microscopy (TEM) images of fresh and frozen EVs (frozen at −80°C) in the presence/absence of 10% DMSO. **(B)** Representative TEM images of the lyophilized EVs (LY EV) in the presence/absence of 10% DMSO. Note that the integrity of frozen EVs and lyophilized EVs is lost in the absence of DMSO.

To evaluate if lyophilization in the presence of DMSO maintains EV membrane characteristics, we observed fresh and lyophilized (10% DMSO and no DMSO) EVs by transmission electron microscopy. Representative images presented in Figure 2A show that, observationally, freezing the EVs in the absence of DMSO affects their integrity. These effects were observed consequently in lyophilized EVs as well (Figure 2B). EVs frozen and lyophilized with 10% DMSO showed morphological similarity to fresh EVs.

### Size distribution, EV marker expression and pro-osteogenic miRNA expression in fresh and lyophilized EVs

**FIGURE 3:**
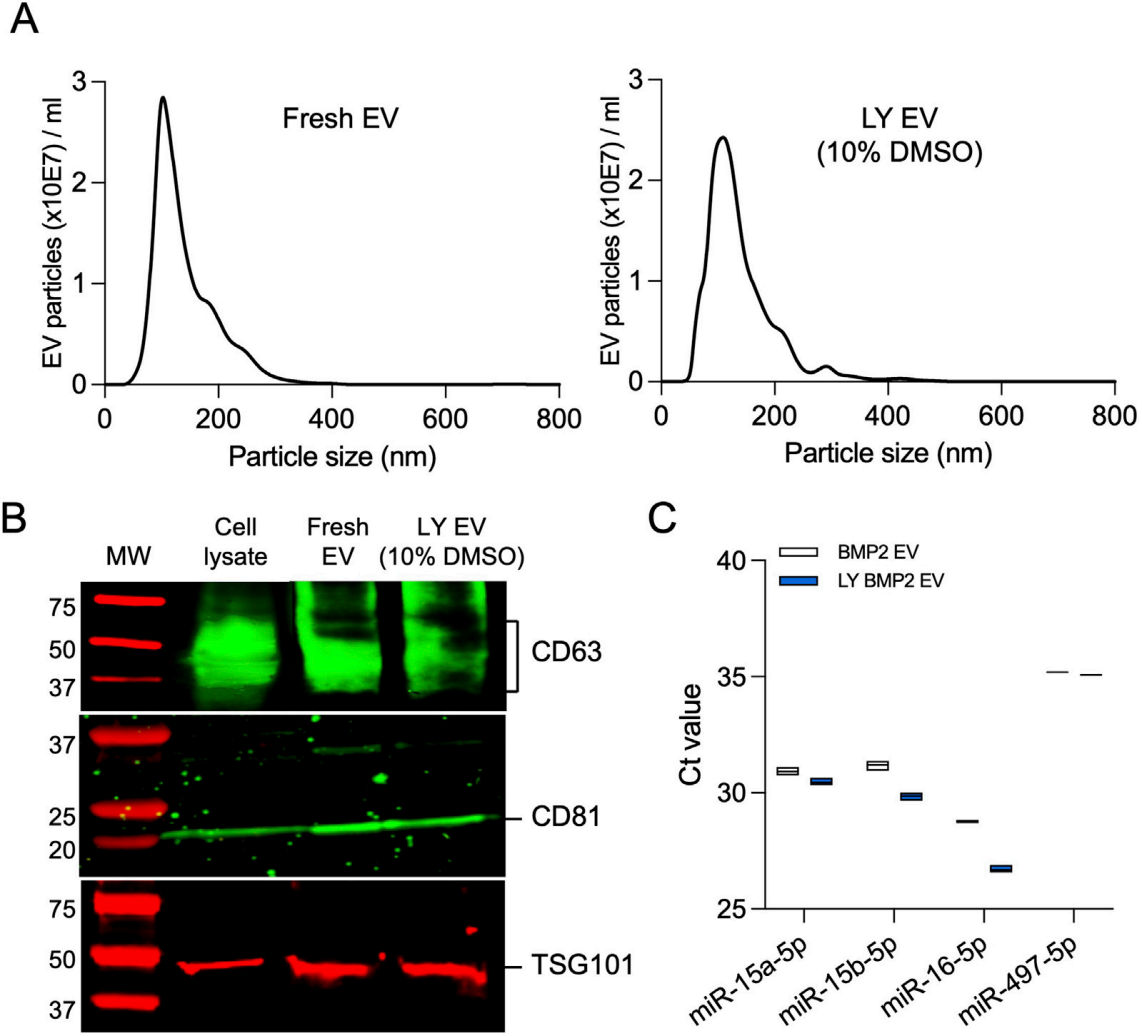
Characterization of lyophilized EVs **(A)** Representative nanoparticle tracking analysis (NTA) plots of fresh BMP2 EVs and lyophilized BMP2 EVs. **(B)** Immunoblot of cell lysate, fresh and lyophilized BMP2 EV for the presence of exosomal marker proteins CD63, CD81 and TSG101. **(C)** Raw Ct value of osteoinducitve miRNAs in fresh and lyophilized BMP2 EVs (n = 3).

We evaluated the size distribution of the EVs isolated fresh and lyophilized with 10% DMSO by NTA (Figure 3A), membrane and intra EV marker expression by immunoblotting (Figure 3B), and the expression levels of osteoinductive miRNAs in BMP2 EVs that are known to bind to the 3′ untranslated region (UTR) of both SMURF1 and SMAD7 (Huang et al., 2020b) by qRT-PCR (Figure 3C). Results presented in Figure 3 indicate that no significant changes were observed in the size distribution or EV marker expression and the expression levels of osteoinductive miRNAs in lyophilized BMP2 EVs remained similar to fresh BMP2 EVs.

### *In vitro* mechanistic and biochemical functionality of lyophilized EVs

**FIGURE 4:**
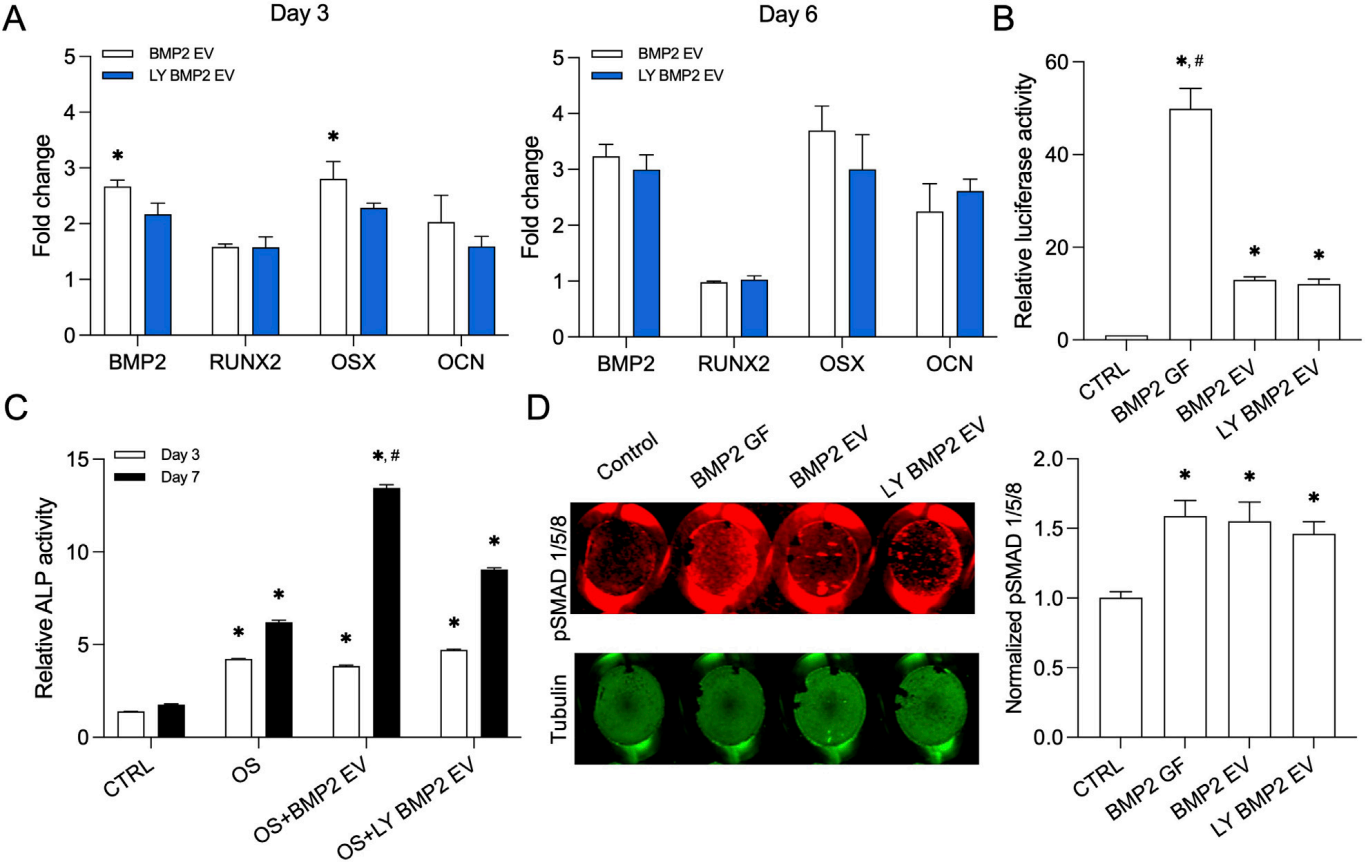
Osteoinductive property of the lyophilized BMP2 EVs **(A)** Osteoinductive gene expression levels in fresh and lyophilized BMP2 EV HMSCs at day 3 and 5 with respect to control (no EV) group. Data are represented as mean fold changes ± SD (n = 4). *: statistical significance with respect to BMP2 EV group as measured by student’s t-test (P< 0.05). **(B)** Graph representing relative luciferase activity in HMSCs transfected with a BMP2 reporter luciferase plasmid (n = 4). Recombinant BMP2 protein (BMP2 GF) was used as positive control. Note the significant increase in luciferase activity in fresh and lyophilized BMP2 EV treated groups compared to control. *: statistical significance with respect to control, #: statistical significance with respect to BMP2 GF group as measured by Tukey’s test post ANOVA (P< 0.05). **(C)** Graph representing relative alkaline phosphatase (ALP) activity in HMSCs treated with osteogenic differentiation medium (OS) in the presence/absence of fresh and lyophilized BMP2 EVs at 3 and 7 days (n = 4). *: statistical significance with respect to control, #: statistical significance with respect to OS + BMP2 EV group as measured by Student’s t-test (P< 0.05). **(D)** Quantitative in-cell western for the presence of phosphorylated SMAD 1/5/8 and the corresponding tubulin expression in a 96 well plate assay (n = 6). The graph represents quantitation of the fluorescence from the wells using the LICOR Odyssey imager normalized to tubulin. *: statistical significance with respect to control group as measured by Tukey’s test post ANOVA (P< 0.05).

We compared fresh and lyophilized (with 10% DMSO) BMP2 EVs for their osteoinductive function. We analyzed the gene expression of osteogenic markers runt-related transcription factor 2 (RUNX2), bone morphogenetic protein 2 (BMP2), osterix (OSX) and osteocalcin (OCN) in naïve HMSCs 3 and 6 days post treatment with the EVs under normal growth conditions. Results presented in Figure 4A show that both fresh and lyophilized BMP2 EVs increased the expression of these genes over control and no significant differences were observed between the activities of fresh and lyophilized EVs (n = 4 for all groups and time points). In published studies, we showed that the BMP2 EVs enhance the promoter activity of a BMP2 specific response element (Huang et al., 2020b). We used the luciferase-based reporter assay here to compare the activities of fresh and lyophilized BMP2 EVs. Recombinant human BMP2 served as the positive control. Results presented in Figure 4B show that while the EVs are not as effective as the growth factor, both fresh and lyophilized EVs showed similar activity and no statistical difference was observed between these groups (n = 4 for all groups). We followed up these experiments by comparing the ability of the EVs to enhance alkaline phosphatase (ALP) activity (Figure 4C). When HMSCs were subjected osteogenic differentiation, ALP activity increased on days 3 and 7 post osteogenic differentiation. This activity was significantly enhanced by BMP2 EVs albeit only at day 7. However, in this experiment, we did observe that at day 7, there was a statistically significant difference between the activity of fresh and lyophilized BMP2 EVs (n = 4 for all groups and time points). To verify whether this effect also translates to other aspects of the BMP2 pathway, we analyzed SMAD1/5/8 phosphorylation in the presence of fresh and lyophilized BMP2 EVs using an in-cell western analysis. The BMP2 EVs downregulate SMAD7 and SMURF1 and enhance SMAD1/5/8 phosphorylation (Huang et al., 2020b). For these experiments, tubulin was used as a control for normalization. Results presented in Figure 4D show that both fresh and lyophilized BMP2 EVs enhanced SMAD1/5/8 phosphorylation with respect to untreated controls and on par with rhBMP2. No statistically significant differences were noted between the EV groups (n = 6 for all groups).

**FIGURE 5:**
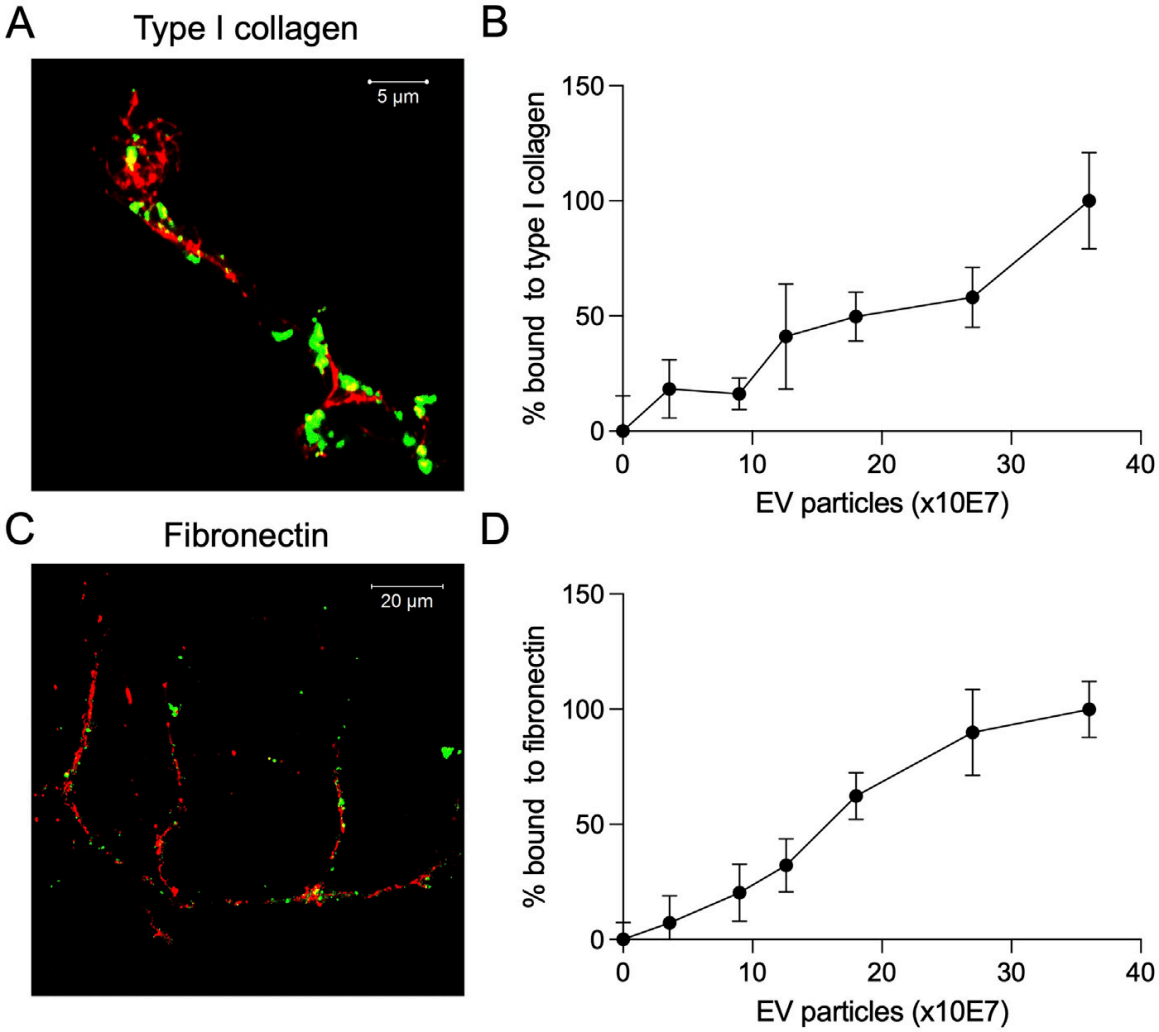
Binding of lyophilized EVs to ECM proteins **(A)** Representative confocal image showing the binding of fluorescently labeled lyophilized EVs (green) to the decellularized ECM of HMSCs. Counterstain was performed for type I collagen (red, scale bar = 5 μm). **(B)** Dose-dependent binding of fluorescently labeled lyophilized EVs to type I collagen coated assay plates (data points represent mean ± SD, n = 6). **(C)** Representative confocal image showing the binding of fluorescently labeled lyophilized EVs (green) to the decellularized ECM of HMSCs. Counterstain was performed for fibronectin (red, scale bar = 20 μm). **(D)** Dose-dependent binding of fluorescently labeled lyophilized EVs to fibronectin coated assay plates (data points represent mean ± SD, n = 6).

MSC EVs and the BMP2 EVs possess integrins on their membrane that enable them to bind dose dependently to ECM proteins such as type I collagen and fibronectin (Huang et al., 2016; Huang et al., 2020b; Huang et al., 2021). Therefore, we evaluated the ability of the lyophilized BMP2 EVs to bind to ECM proteins. We qualitatively evaluated this ability using decellularized ECM from HMSCs. Fluorescently labeled lyophilized BMP2 EVs were incubated with decellularized HMSC ECM and counterstained for type I collagen and fibronectin (Figures 5A, C). Representative confocal images show a co-localization of the EVs (green) with the respective ECM proteins (red). Quantitative analyses were performed using fluorescently labeled EVs in 96 well plates coated with type I collagen and fibronectin. Results presented in Figures 5B, D show dose dependent binding of the lyophilized EVs to these proteins (n = 6 for all time points). Overall, these mechanistic studies indicated that the lyophilized EVs performed on par with fresh BMP2 EVs in all aspects except for a loss of activity observed in the ALP assay.

### *In vivo* functionality of the lyophilized EVs

**FIGURE 6:**
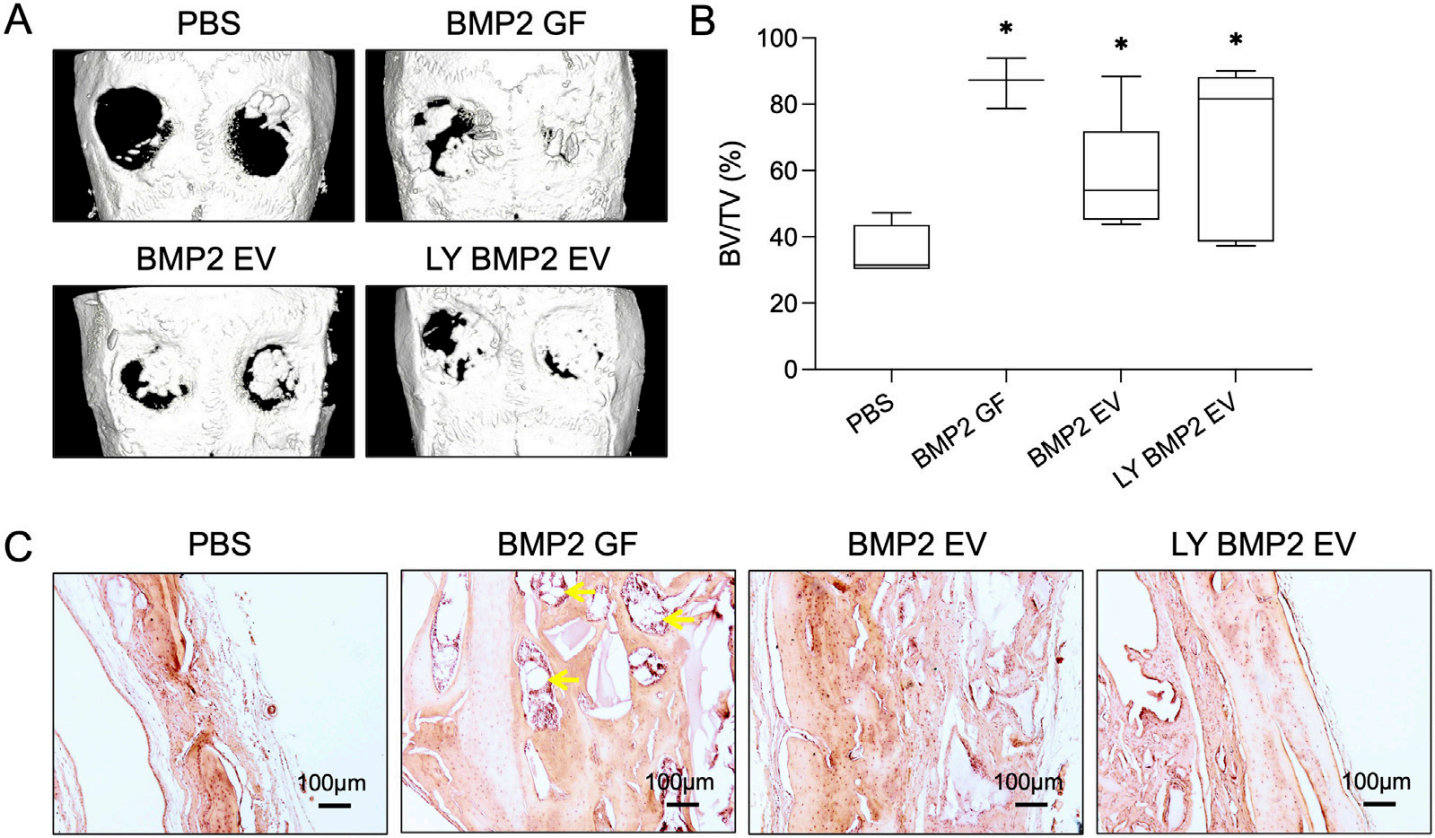
*In vivo* osteoinductive properties of the lyophilized EVs **(A)** Representative μCT images showing bone regeneration 5 mm rat calvarial defects in the presence/absence of fresh and lyophilized BMP2 EVs after 8 weeks post wounding. Recombinant BMP2 protein (BMP2 GF) was used as positive control. **(B)** Volumetric quantitation of bone formation in the defects (n = 8). *: statistical significance with respect to the control group as measured by Tukey’s test post ANOVA (P< 0.05). **(C)** Representative light microscopy images of decalcified calvaria sections with H&E staining. Scale bar = 100 μm. The yellow arrows in the BMP2 GF group point to fat deposits within the regenerated bone.

Following up from the *in vitro* studies, we evaluated the ability of the fresh and lyophilized BMP2 EVs *in vivo* in a rat calvarial defect model. The EVs were encapsulated in clinical grade collagen sponge for these experiments. Figure 6A shows representative μCT images of the rat calvaria 8 weeks post implantation. Observationally, both fresh and lyophilized BMP2 EVs showed similar levels of bone regeneration in the defects (Figure 6B). Volumetric quantitation of the μCT data showed that both EV groups were significantly better than the control group (collagen sponge with PBS). No statistical significance was observed between the fresh and lyophilized EV groups (n = 4 for all groups). As noted in our previous publications, while rhBMP2 shows volumetric bone, the nature of the bone is spongy with large pockets of fatty marrow when observed histologically (yellow arrows in Figure 6C). On the other hand, the BMP2 EVs promote bone regeneration that histologically resembles native bone. This is evident in the representative images of H&E stained sections presented in Figure 6C.

Overall, the *in vivo* results showed that both fresh and lyophilized BMP2 EVs showed similar osteogenic and osteoinductive capabilities.

## Discussion

The focus of this manuscript is on the mechanistic functionality of lyophilized EVs. This aspect of EV function, especially for engineered EVs have not been thoroughly investigated in published literature. Specifically, there is a significant gap in knowledge from an *in vivo* perspective. Considering this, throughout this manuscript, we performed studies using functionally engineered EVs (FEEs) that enhance bone repair via augmenting the BMP2 signaling cascade (BMP2 EVs). Our laboratory has generated and characterized these EVs extensively and therefore, they served as an ideal platform for this study (Huang et al., 2020b; Huang et al., 2021). Therefore, this manuscript focused on comparing fresh and lyophilized FEEs and not on establishing FEE functionality.

As a cryoprotectant, we used DMSO in this study. DMSO has been used as a pharmacological agent since the 1960s and is presently used as a cryoprotectant for storage of stem cells. Additionally, it is also used for treatment of musculoskeletal and dermatological diseases as well as for relieving of cranial pressure with minimal side effects (reviewed in (Kollerup Madsen et al., 2018)). These evidence and the wide usage of the agent in current drugs prompted us to evaluate its suitability as a cryoprotectant for EVs. It is possible that DMSO alters some EV properties to render its protective role. However, this study focused on the EV functionality and its applicability for *in vivo* use and not specifically on the molecular/structural changes that DMSO may elicit on EV membranes. Because the cryopreserved and lyophilized EVs maintained their functionality, it is possible to extrapolate that the effects of DMSO on the EVs did not significantly alter its functional significance. Published studies on EV lyophilization have used various sugars such as sucrose and trehalose for EV cryoprotection as well. While this study focuses on the applicability of DMSO, it does not discount the functionality of other cryoprotectants. Further studies are required to validate the functionality of EVs lyophilized in their presence to ensure that the physical protection they confer translates to functional protection as well.

The results presented in Figures 1–3 collectively show that during the process of freeze drying, the EVs can undergo dramatic changes that affect their function. We began by evaluating the endocytic efficiency of the EVs in the presence and absence of DMSO. Endocytosis is the first necessary step for EV bioactivity. Loss of this function prevents transfer of EV cargo within recipient cells and as a result, loss of activity. We were able to observe a dose-dependent effect of DMSO on EV endocytosis both quantitatively and qualitatively. We began the study by first establishing a concentration of DMSO that preserved the EV endocytic ability. Based on these results and having identified 10% DMSO as a suitable concentration for freezing and lyophilizing EVs, we set out to test the lyophilized EVs for their functional efficacy.

From this perspective, we proceeded to evaluate if the EVs showed any changes in physical characteristics. We did not see any meaningful differences in the NTA experiments for EV size distribution between fresh and lyophilized EVs and immunoblotting showed expression of EV markers intact in both types of EVs. Electron microscopy analysis of fresh and lyophilized EVs (with 10% DMSO) showed intact EVs. In the absence of DMSO, we observed EV membrane damage in a considerable number of EVs. We were unable to determine the percentage of EVs that showed this type of damage due to the limitations of the qualitative approach (TEM). Even a semi-quantitative evaluation using TEM images will yield results that are applicable only to a fraction of the EV population and will not represent the whole spectrum of the samples. But the images serve to show the type of damage lyophilization can inflict on unprotected EVs. Taken together with the endocytosis data that showed that EVs lyophilized in the absence or reduced presence of DMSO (5%) showed loss or significant reduction in endocytic ability by HMSCs, it is possible to extrapolate that unprotected EVs undergo significant physical damage during the process of lyophilization and this may affect their endocytic efficiency as well as cargo integrity. EVs protected with 10% DMSO before lyophilization not only showed a similar endocytic profile to that of fresh EVs, but also showed similar reduction in endocytic efficiency in the presence of inhibitors heparin and reduced temperature.

In our published work, we have shown that MSC EVs are endocytosed via the cell surface HSPGs (Huang et al., 2020a). Heparin mimics this interaction and pretreatment of EVs with heparin significantly reduces their ability for HSPG binding. This is reflected in reduced overall endocytic efficiency quantitatively. The process of EV endocytosis is also ATP dependent and reduction in temperature significantly affects endocytic efficiency (Huang et al., 2020a). In results presented in this study, both fresh and lyophilized EVs showed a similar reduction in endocytosis with these inhibitors, indicating that lyophilization with DMSO does not alter the EV endocytic pathway.

The BMP2 EVs used in this study possess the ability to induce osteogenic differentiation of MSCs. This is achieved by a cluster of miRNAs in the EVs that negatively regulate SMAD7 and SMURF1. SMAD7 and SMURF1 are negative regulators of the BMP2 cascade. This creates a positive feedback loop that triggers SMAD1/5/8 phosphorylation and the activation of downstream signaling cascade (Huang et al., 2020b). Using a variety of pathway specific assays, that include upstream and downstream targets of the BMP2 signaling cascade, the results presented in this study collectively show that the a cluster of osteoinductive miRNAs are preserved in the lyophilized BMP2 EVs and function on par with that of fresh EVs in triggering osteogenic differentiation of naïve HMSCs, inducing SMAD 1/5/8 phosphorylation, increasing the alkaline phosphatase activity and triggering the activity of BMP2 pathway specific response element in a promoter driven luciferase activity assay. This important finding bears functional significance to the translation of EV therapeutics in showcasing the mechanistic functionality of the lyophilized EVs. From these results, that showed cargo intactness within lyophilized EVs, it is also possible to extrapolate that the EV cargo is protected by maintaining the EV membrane integrity using a cryoprotectant during lyophilization. However, further studies are required with detailed proteomics and other types of omics-based approaches to evaluate if all types of EV cargo remain similar between fresh and lyophilized EVs.

EVs possess integrins on their membrane that enables them to attach to extracellular matrix proteins such as type I collagen and fibronectin (Huang et al., 2016; Huang et al., 2021). This inherent property is a key to their functionality in site-specific regenerative medicine applications. As lyophilization significantly affects membrane characteristics of EVs, it is important for lyophilized EVs to maintain this property. We compared the ability of fresh and lyophilized BMP2 EVs to bind to cell-secreted collagen and fibronectin. Qualitative and quantitative results presented here show that both fresh and lyophilized EVs show similar collagen and fibronectin binding properties. Based on these *in vitro* observations, we proceeded to investigate the ability of the fresh and lyophilized EVs to enhance bone regeneration *in vivo.* For these rat calvarial defect experiments, we used a clinical grade collagen sponge as a carrier for the EVs. Results from these studies showed that both fresh and lyophilized BMP2 EVs enhanced bone regeneration in a similar fashion in concurrence with the published functionality of these EVs (Huang et al., 2020b; Huang et al., 2021). Quantitatively, there was no significant difference between these two groups while significance was observed with respect to the control group. The bone formation from volumetric perspective was not as high as that of the rhBMP2 growth factor, but the fatty marrow that is associated with the rhBMP2 induced bone that is clearly visible in histology was not observed in either of the EV groups confirming our previous observations on the ability of these engineered EVs to induce the formation of bone on par with the histological properties of natural bone.

## Conclusion

Overall, this study highlights that engineered MSC EVs can be lyophilized by using DMSO as a cryoprotectant without loss of mechanistic and functional properties both *in vitro* and *in vivo*. These studies are an important step towards the translation of EV therapeutics, and they fill an important knowledge gap regarding the usability of lyophilized EVs in regenerative medicine. These studies, however, represent only a data point in the spectrum of studies required to show the applications of lyophilized EVs. Future work by us and others can focus on the long-term stability of these EVs, their applicability to various tissues and delivery mechanisms, as well as important comparative studies using different cryoprotectants that have been published recently.

## Data Availability

The raw data supporting the conclusions of this article will be made available by the authors, without undue reservation.
